# HELPING SENIORS AGING IN PLACE: ADULT GUARDIANSHIP AND ITS ROLE IN GERIATRIC SOCIAL WORK PRACTICE IN CHINA AND JAPAN

**DOI:** 10.1093/geroni/igad104.2898

**Published:** 2023-12-21

**Authors:** Zi Yan

**Affiliations:** Waseda University, Tokyo, Tokyo, Japan

## Abstract

As the most aging society in East Asia, a renewed adult guardianship system for the idea of respect for self-determination, practical use of remaining abilities, and re-socialization have been practiced in Japan for 20 years. While, compared with Japan, in lieu of a comprehensive adult guardianship regime, not until 2017, voluntary guardianship notarization has become the main alternative for supported decision-making and self-determination for incapacitated adults and a tool for mitigating the social risks of the frail elderly in China. Based on secondary data and semi-structured interviews, this study is the first attempt that demonstrates a comprehensive comparison of the recent developments and current forms of adult guardianship initiatives in mainland China and Japan. Besides highlighting the difficulties and challenges faced by these two countries, this article also outlined the types of guardians under their current legal framework, identified the social functions of guardians, the notary office in adult guardianship socialization, and the emerging geriatric social work initiatives adopted. The geriatric social work practices in China and Japan’s adult guardianship point in particular toward the potential provocativeness: adult guardianship in the eastern context—a context marked by a strict family system that emphasizes the virtues of filial piety and discourages outside intervention in family matters. As the number of people with dementia is projected to reach 82 million in 2030, China and Japan’s experience potentially holds wide significance for countries that lack substantial policies and programs for caring for the frail elderly.

